# Patients’ Preferences for Bone-Anchored Prostheses After Lower-Extremity Amputation: A 2-Center Discrete Choice Experiment in The Netherlands (PREFER-BAP-1)

**DOI:** 10.2106/JBJS.24.00204

**Published:** 2024-09-06

**Authors:** Gabriel-Kyrillos M. Saleib, Marcel F. Jonker, Mark G. Van Vledder, Michael H.J. Verhofstad, Maria A. Paping, Ruud A. Leijendekkers, Oscar J.F. Van Waes

**Affiliations:** 1 Trauma Research Unit, Department of Surgery, Erasmus MC, University Medical Center Rotterdam, Rotterdam, The Netherlands; 2 Erasmus Choice Modelling Centre, Erasmus University Rotterdam, Rotterdam, The Netherlands; 3 Erasmus School of Health Policy & Management, Erasmus University Rotterdam, Rotterdam, The Netherlands; 4 Osseointegration Center Rotterdam, Rotterdam, The Netherlands; 5 Rijndam Rehabilitation, Rotterdam, The Netherlands; 6 Orthopedic Research Laboratory, Radboud University Medical Center, Nijmegen, The Netherlands; 7 Department of Rehabilitation, Radboud University Medical Center, Nijmegen, The Netherlands; 8 Radboud Institute for Health Sciences, IQ Healthcare, Radboud University Medical Center, Nijmegen, The Netherlands

## Abstract

**Background:**

The rising popularity and use of a bone-anchored prosthesis (BAP) involving an osseointegrated implant for patients with lower-limb amputations experiencing socket-related issues have led to increased interest in the measurement of clinical and functional outcomes. However, the value of BAP treatment characteristics from the patient perspective has not yet been investigated. This study aimed to determine the relative importance of specific BAP characteristics, and the effect of complications in quality-of-life (QoL) points and monetary utility decrement (loss [€]), using a 2-center discrete choice experiment (DCE) conducted in The Netherlands.

**Methods:**

A DCE was developed that included the most salient characteristics of BAP treatment based on a review of the literature and qualitative and quantitative methods. The following characteristics were selected: QoL change, short- and long-term complications, osseointegrated implant survival, and out-of-pocket contributions (costs). Patients aged 18 to 99 years who were eligible for, or had already received, an osseointegrated implant were invited to participate, after informed consent, to elicit BAP treatment preferences. A Bayesian mixed logit model was used.

**Results:**

Two hundred and forty-seven completed surveys were collected; 64% of the patients were male, 73% had undergone a transfemoral amputation, and 33% had >36 months of experience with a BAP. Patients considered long-term complications and QoL the most important characteristics. Long-term complications were 3.4 times more important than short-term complications. Opting out was undesirable, and patients valued better and beneficial levels (associated with better outcomes) of BAP characteristics positively. Implant removal was the level with the greatest loss among all complications, at 1.15 (95% credible interval [CI], 0.96 to 1.38) QoL points and €16,940 (95% CI, €14,780 to €19,040) loss.

**Conclusions:**

To our knowledge, this is the first study to use a DCE to elicit patients’ preferences regarding BAP treatment, outcomes, and related complications; we found that patients strongly care about long-term complications. The results suggest that osseointegrated implant teams and policy-makers should consider these areas when proposing treatment protocols. Furthermore, policy and clinical guidelines for BAP treatment could be enhanced by our results with respect to patients’ perspectives, management of patients’ expectations, and associated losses in QoL points and monetary loss secondary to complications.

**Level of Evidence:**

Therapeutic Level II. See Instructions for Authors for a complete description of levels of evidence.

There is increasing evidence that the use of a bone-anchored prosthesis (BAP) offers long-term clinical advantages over the use of a conventional socket prosthesis (SP) in patients with lower-limb amputation and unresolvable socket-related issues^1-3^. An SP fits over the interposing soft tissue around the residual limb, and thus socket-related issues, such as pressure spots, shear forces, and wounds, are inherent to the SP design. However, with a BAP, the prosthesis is transcutaneously attached directly to the residual limb’s bone utilizing an intramedullary implant, that is, an osseointegrated implant (Fig. 1)^4,5^. Clinical studies have demonstrated favorable outcomes of patients with a BAP compared with a pre-BAP health state in domains of the World Health Organization International Classification of Functioning, Disability and Health (WHO ICF) model regarding body function and structures, and activities and participation, as well as in surgical and rehabilitation outcomes^6-10^,^37^.

**Fig. 1:**
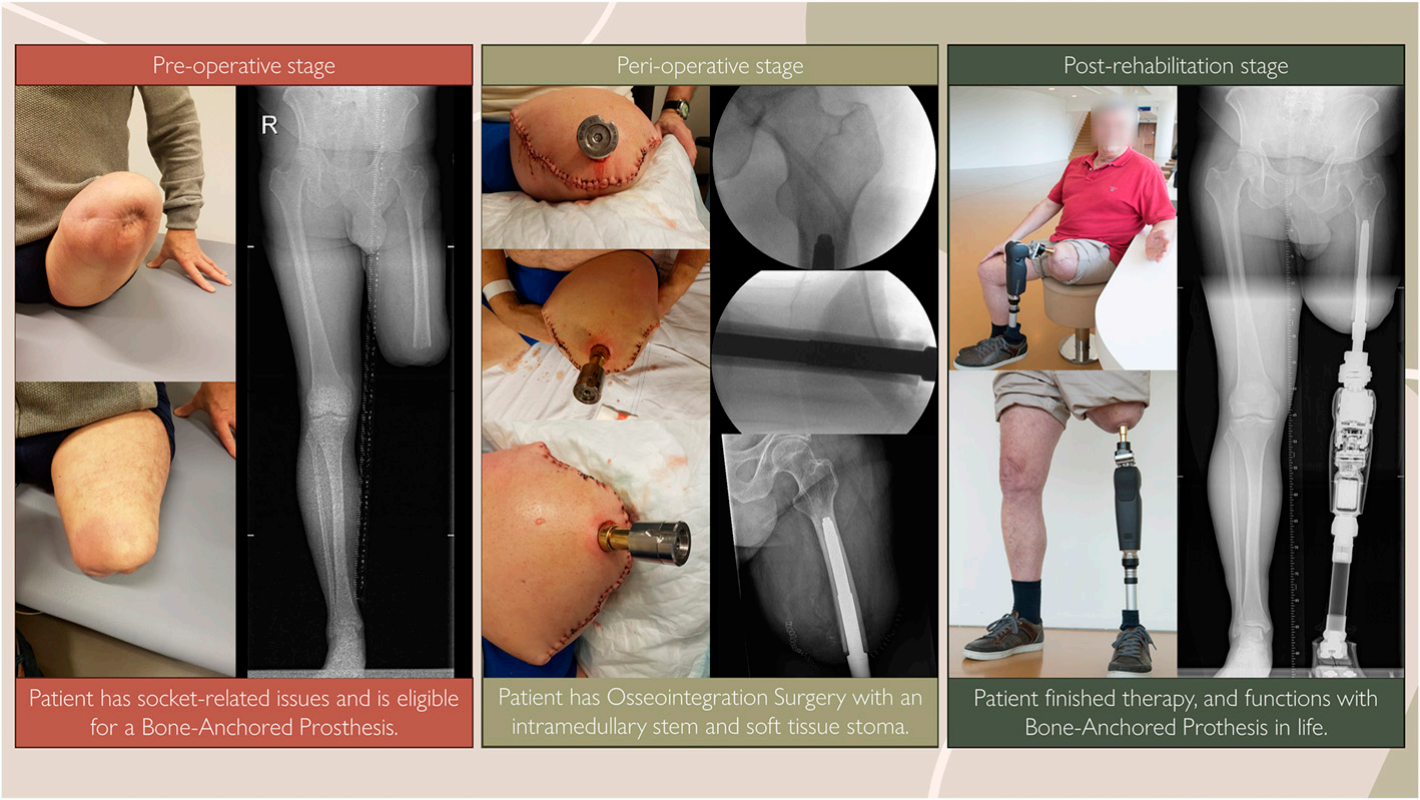
Different stages of bone-anchored prosthesis treatment over time and as shown on radiographic imaging.

In line with the maturation and progress of BAP treatment, patient involvement and elicitation of the decision-maker’s preferences are of utmost importance in the context of shared decision-making, patient-centered and tailored care, and ethical and legal codes^11-14^. Patients’ preferences hold intrinsic value in shaping the provision of patient-centered health care^15^,^16^. Additionally, there is a need to explore how BAP characteristics relate to aspects beyond quality of life (QoL) as generally measured by surveys and instruments^17,18^. Lastly, national health-care policies emphasize the importance of patient involvement, shared decision-making, and the active engagement of stakeholders throughout the health-care continuum^19^.

Therefore, this study complements current knowledge with research assessing patients’ preferences^16,20^. Patients value specific characteristics, complications, and outcomes of surgical interventions differently, which may influence the ideal treatment choice. Stated-preference data are crucial for patient-centered care and understanding the value that patients with lower-extremity amputation and socket-related issues place on processes and outcomes of BAP treatment. With this study, we aimed to evaluate patient preferences for a BAP by quantifying the relative importance of treatment characteristics. We also examined patients’ stated choices with regard to potential complications related to BAP treatment, expressing the associated utility decrements (from here on, called “losses”) in QoL points and monetary terms.

## Materials and Methods

### Theoretical Foundation

To obtain patient preferences, a discrete choice experiment (DCE) was developed following the international standards for recommendations pertaining health economics (see Appendix S1)^21,22^. This methodology was selected to quantify BAP treatment characteristics and trade-offs between characteristics. A DCE is a well-established, but relatively unknown, Nobel prize-winning methodology that can quantify patients’ preferences for treatment characteristics^23^. In DCEs, medical interventions are described on the basis of their characteristics (in DCE jargon, called “attributes”). BAP characteristics represent the different features of the intervention, and each characteristic (e.g., QoL change) can comprise multiple variations, which are called “levels” (e.g., no difference, 1-point increase, 3-point increase). Patients in DCEs express their preferences by repeatedly making choices among hypothetical treatment options, which are created by selecting 1 of the levels of each included characteristic. By consistently choosing among combinations, patients reveal the relative importance they place on the different levels, and numerical values are derived from statistical models, grounded in random utility theory^24,25^. The methodology assumes that patients aim to maximize the benefits derived from their choices, thereby providing insight into the trade-offs they make between different levels.

### DCE Characteristics and Levels

The characteristics and levels (Table I) of this DCE were selected through an extensive, iterative process that comprised literature reviews^1-4,7,9,26-29^ plus exploratory interviews and meetings with stakeholders (expert multidisciplinary teams), including former and current patients (n = 10), surgeons (n = 2), rehabilitation doctors (n = 2), physiotherapists (n = 2), researchers (n = 2) with clinical, epidemiological, and stated-preference expertise, a nurse, an orthopaedic instrument maker, and a trauma physician assistant. Subsequently, face-to-face interviews following a think-aloud protocol and information saturation were conducted with other patients with a BAP (n = 5). Iterative evaluations were conducted to assess the relevance and graphical presentation of the characteristics and their levels, and the clarity of the level definitions. The characteristics identified as most relevant were QoL change, short- and long-term complications, osseointegrated implant survival, and out-of-pocket contributions (costs).

**TABLE I:** Characteristics and Levels of the Discrete Choice Experiment*

Characteristic	Characteristic Description	Level	Level Description
QoL change	QoL change compared with situation before BAP, on a scale from 0 to 10. Patient’s starting point does not matter, and all steps are equal in effect	3-point increase	Increase of 3 points in QoL after OI surgery compared with situation before BAP
		1-point increase	Increase of 1 point in quality of life after OI surgery compared with situation before BAP
		No difference	There is no difference in quality of life before and after OI surgery (reference level)
		1-point decrease	Decrease of 1 point in quality of life after OI surgery compared with situation before BAP
Short-term complications	In the first year, intensive follow-up is provided, in part because of complications that could occur. Patients are made aware of what complications could occur, and the chances, and how they are resolved or treated	No major complications	No complications or adverse events that need therapy or follow-up (reference level)
		33% stoma infection, nonsurgical therapy	33% chance of having a low-grade soft-tissue infection, which can be resolved using oral antibiotics
		15% stoma infection, surgical therapy	15% chance of having a soft-tissue infection, which only can be treated with a surgical intervention
		6% hypergranulation	6% chance of having hypergranulation, which is excessive granulation that rises above the wound surface or stoma, and needs debridement
		6% bone fracture	6% chance of having a bone fracture (periprosthetic fracture)
Long-term complications	Complications that could occur after the first year and up to one’s lifetime. No chances are given as no time period is provided.	No complications	No complications or adverse events that need therapy or follow-up (reference level)
		Bone infection	Bone infection (osteomyelitis)of any sort, that can be resolved without surgery
		Implant removal	Surgical removal of the osseointegrated material
		Chronic stoma problems	Chronic stoma problems that need regular check-up, visits, and treatments
		External component replacement	External component replacement including a visit to the instrument maker. External components are the dual-cone adaptor, height or gait adjustment of the outer mechanism, or anything that the instrument maker can change or fix
OI implant survival	It is unknown how long the implant can survive or will get loose. Knowing this information, what is the least amount of time that is still accepted by patients?	5 yr	5 yr (reference level)
		10 yr	10 yr
		20 yr	20 yr
Out-of-pocket-contributions	BAP treatment is expensive; if full reimbursement is not possible, how much is a patient willing to pay to have BAP treatment?	No contribution	No personal monetary contribution (reference level)
		€5,000	A contribution of €5,000
		€10,000	A contribution of €10,000
		€15,000	A contribution of €15,000
		€20,000	A contribution of €20,000
		€25,000	A contribution of €25,000
No BAP	Patient does not want BAP treatment	Opting out	Opting out of BAP treatment and thus staying at status quo

* These are the characteristics and levels for osseointegrated implant (OI) options in a choice task. Reference levels were utilized as the base level for the statistical analysis. BAP = bone-anchored prothesis, and QoL = quality of life.

### DCE Design

Assessing all possible combinations of the included levels would result in a prohibitively large experimental design. To reduce this to a more manageable subset, an orthogonal design with 21 “choice tasks” per respondent was generated and fielded to a pilot sample of 20 respondents. Preference estimates obtained from the pilot sample were used to generate a more efficient Bayesian DCE design with 10 subdesigns and 21 choice tasks per respondent. The design optimizations were performed using Spotlight (https://spotlight-software.com), and the efficiency criterion was calculated as the weighted average Bayesian D-error for a main-effects conditional logit model, with 90% of the weight assigned to the average of the individual subdesigns and 10% to the efficiency of the overall design. Using a weighted design criterion ensured that not too much individual-level design efficiency was foregone to achieve a marginally higher overall design efficiency. To maximize statistical efficiency, the design was updated every 20 to 30 respondents with improved Bayesian priors based on the observed choices. Each respondent was randomly assigned to only 1 of the DCE design versions.

### Patient Recruitment, Data Collection, and Ethics

All patients 18 to 99 years of age from the Osseointegration Center Rotterdam (OCR) in Rotterdam and Radboud University Medical Center (RUMC) in Nijmegen, The Netherlands, who either were on the waiting list for BAP surgery or had already received an osseointegrated implant, and who could provide informed consent, were considered eligible for study inclusion. OCR is a a collaboration of the Trauma Research Unit Department of Surgery at Erasmus MC, Rijndam Rehabilitation, and Rijndam Orthopedic Technic, all institutions in Rotterdam, The Netherlands. OCR utilizes only the Osseointegration Prosthetic Limb (OPL; Permedica) system. RUMC uses the following implants: the OPL, the Integrated Leg Prosthesis (Orthodynamics), and the Bone Anchoring Device for Artificial Limbs (OTN Implants). Individuals with insufficient knowledge of the Dutch language were excluded. Eligible patients were contacted, and written or digital informed consent was obtained. A personalized digital link with the survey, which included the DCE, was then sent via email. After 3 weeks, nonresponders were sent a reminder, and after 5 weeks, were called. All data were collected from January 2022 to June 2023. Incomplete surveys and surveys finalized in <10 minutes were removed. This study was conducted in accordance with the Declaration of Helsinki (October 2013) and was approved by the Medical Ethical Committee of Erasmus MC, University Medical Center Rotterdam (MEC-2022-0068).

### Survey Development and Structure

The survey was created in Dutch level B1 (intermediate proficiency and language understanding), and was fielded using Lighthouse Studio (https://sawtoothsoftware.com). The survey was piloted through interviews (n = 20) to check for any problems in interpretation, survey length, cognitive burden, and face validity. The survey consisted of 4 sections. The first section presented an informed-consent statement that respondents had to agree to before being able to proceed with the remainder of the survey. In the second section, the BAP characteristics and levels were explained one-by-one, alternating with practice discrete choice tasks that included the previously discussed characteristics and levels. This warm-up process gradually increased the complexity of the choice tasks, progressively familiarizing respondents with the trade-offs and the visual layout of the choice tasks. In the third section, the 21 choice tasks were presented to respondents in 3 blocks of 7 questions, with the EuroQol-5 Dimensions-5 Levels (EQ-5D-5L) questionnaire^30^ and several demographic, treatment-related, and socioeconomic-related questions in between. The choice tasks were formatted with level overlap and color coding^31^ and presented to respondents in a so-called dual-response-none format, in which patients initially had to choose between 2 BAP treatment options (Fig. 2-A) and then answered a follow-up question in which the previously chosen option was displayed with a “no” option (“I would not choose the option”) (Fig. 2-B). Opting for the “no” option meant that the patient, in that scenario, would not consider a BAP given the 2 options involving a BAP. In the closing section, evaluation and cognitive debriefing questions (on a 5-point Likert scale) about the survey and the BAP treatment were shown. Upon completion of these tasks, respondents received a survey completion page with an open-ended text question for any additional comments or feedback. The (Dutch) survey is available from the first author (G.-K.M.S.) on request.

**Figs. 2-A and 2-B** Example of a choice task question in the discrete choice experiment. BAP = bone-anchored prosthesis, and OI = osseointegrated. The figures are representative of 1 choice task set (**Fig. 2-A**) and the follow-up choice task in which the patient is asked whether they would prefer the previously chosen option versus the “no” option (i.e., no BAP treatment) (**Fig 2-B**). Characteristic levels that are equal (e.g., in Fig. 2-A, “Expected survival of OI Implant” and “10 years”) are grayed out, and higher-level orders of a characteristic (e.g., in Fig. 2-A, “A contribution of 25,000 euro” being a greater out-of-pocket cost than “A contribution of 20,000 euro”) are shown in a darker shade of purple. Coloring styles are used to reduce [cognitive] burden. The levels within each characteristic change from one choice task set to the next. Note that the choice task and follow-up choice task are displayed on consecutive pages.

**Fig. 2-A:**
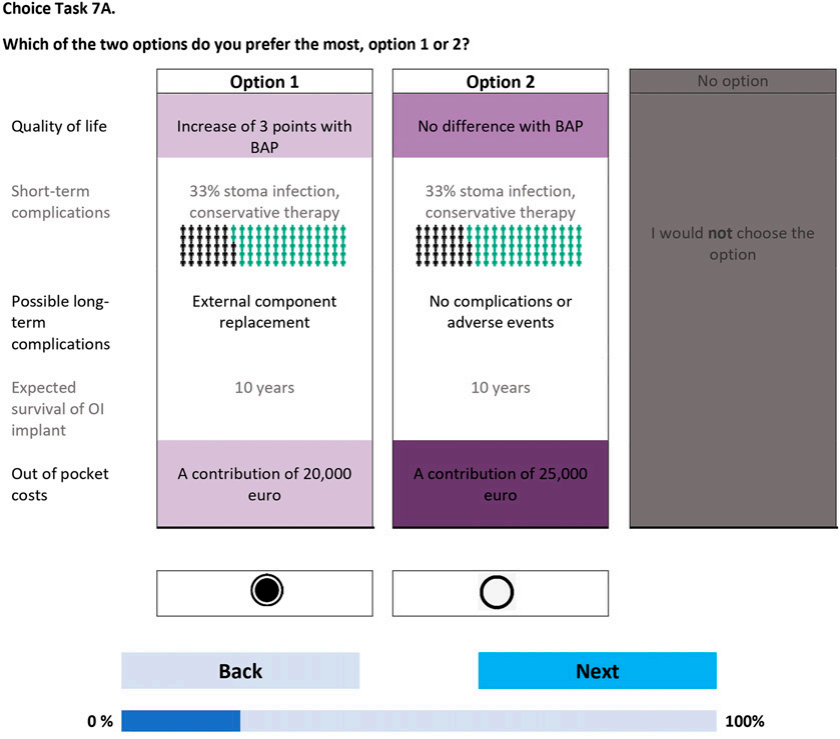

**Fig. 2-B:**
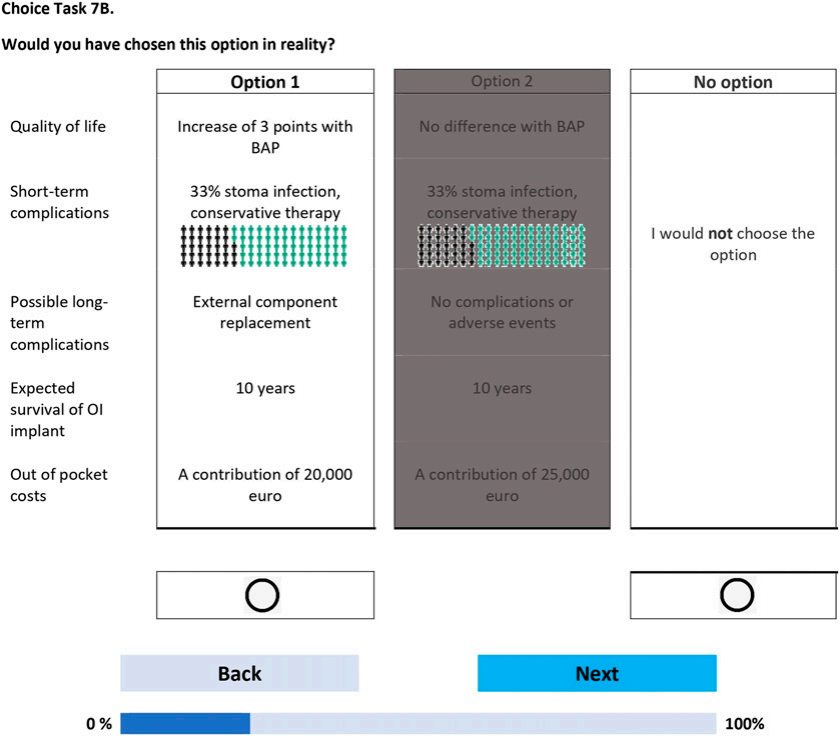

### Statistical Analysis

In keeping with best-practice methods, the DCE choice data were analyzed using a mixed logit (MIXL) model (see Appendix S1)^32^. More specifically, a Bayesian-panel MIXL model was fitted to obtain estimates of the respondents’ mean preference weights, relative importance of BAP characteristics, and complications expressed in monetary (i.e., euros; €1 = $1.06; 95% confidence interval, $1.06 to $1.07) and in QoL point loss, while appropriately accounting for the choice data panel structure and for unobserved differences in preferences across respondents (i.e., preference heterogeneity)^33^. BAP characteristics were dummy coded in the MIXL model. Although the estimates are obtained on a latent scale that is not directly interpretable, the relative magnitude and sign of the parameters reflect the relative importance and direction of the levels compared with the base case (i.e., more or less preferred than the base-case level), respectively.

Based on the (level) preference weights, relative treatment characteristics’ importance weights were calculated as the maximum change in preference weights that can be achieved with the levels for each characteristic. Numeric values of the loss in QoL and euros for complications were linearly interpolated from the QoL and out-of-pocket-contribution characteristics, accounting for potentially nonlinear preferences^34^.

The MIXL model was implemented in the BUGS language and estimated with the OpenBUGS software package using Bayesian Markov chain Monte Carlo (MCMC) methods. A total of 75,000 draws from 3 independent MCMC chains were used to reliably approximate the posterior distribution, with an initial 25,000 draws discarded as burn-in iterations. Convergence was assessed through visual inspection of the chains and the diagnostics as implemented in the OpenBUGS software. The BUGS model code and prior distributions’ specification are provided in Appendix S2. A full explanation, both mathematical and in plain English, of the DCE methodology and the statistics is provided in Appendix S3.

## Results

### Patient Sample

**Fig. 3:**
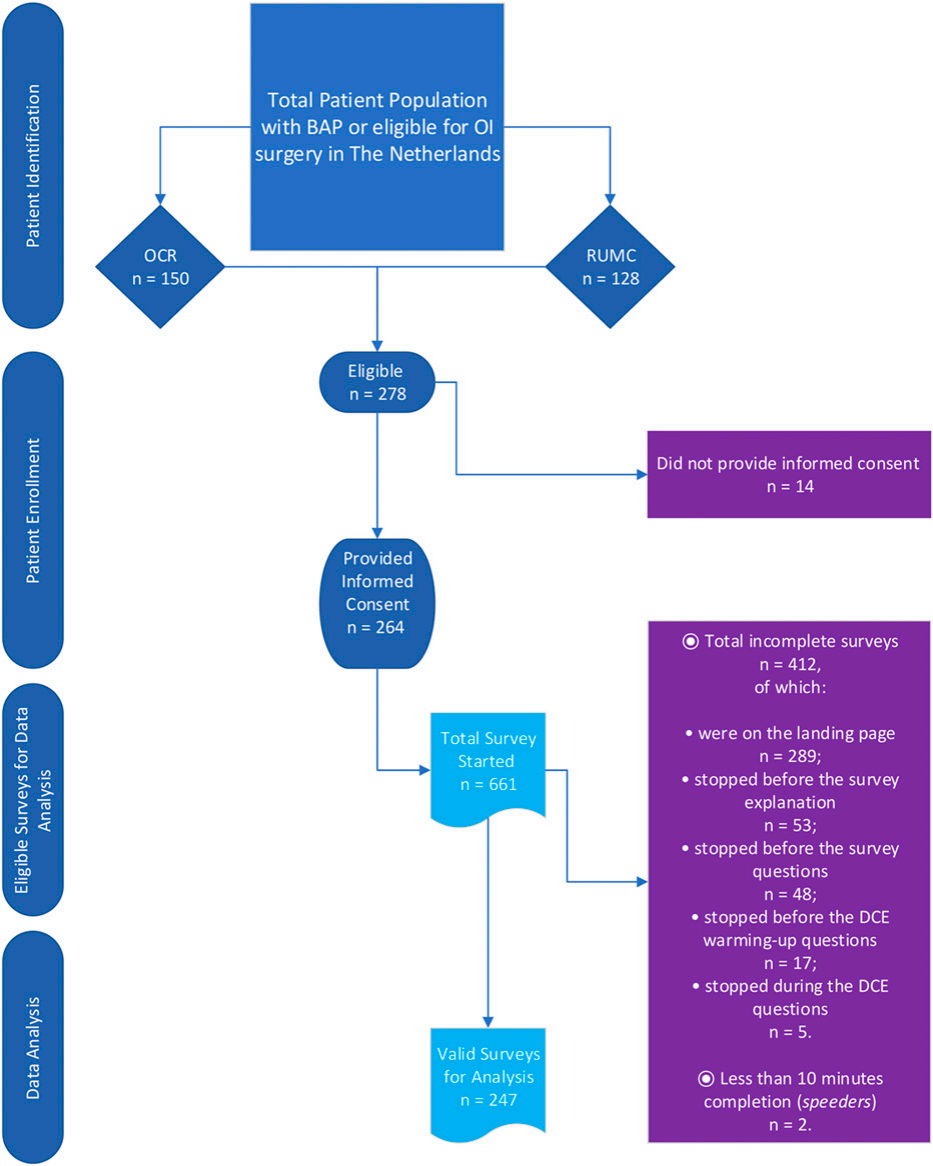
Study flowchart. Note that 264 patients provided informed consent; however, patients may have opened the survey, which was sent by e-mail with a personal link, multiple times resulting in a high number of surveys started (n = 661). However, only completed surveys were used for data analysis (n = 247). Reasons for incomplete surveys are provided in the purple exclusion box. BAP = bone-anchored prosthesis, OI = osseointegrated implant, OCR = Osseointegration Center Rotterdam, RUMC = Radboud University Medical Center, and DCE = discrete choice experiment.

Two hundred and seventy-eight patients (Fig. 3) met the inclusion criteria (OCR, n = 150; RUMC, n = 128), of whom 264 provided informed consent and participated in the survey. Two hundred and forty-nine completed the survey, with an average and median survey completion time of 65 and 38 minutes, respectively. Ninety-nine percent of the respondents completed the survey in ≥10 minutes. Two respondents who spent <10 minutes were identified as “speeders” and removed from the sample. This resulted in 247 participating patients in the final sample.

Of the 247 patients, 159 (64%) identified as male. The largest number of patients were in the age categories of 50 to 59 years (n = 75; 30%) and 60 to 69 years (n = 74; 30%) (Table II). The largest number of patients had a left-sided transfemoral amputation (n = 95; 38%), and trauma was the main cause (n = 156; 63%). Eighty-two (33%) had >36 months of experience with a BAP. Twenty-six (11%) of the patients were on the waiting list for BAP surgery.

**TABLE II:** Patient, Amputation, and BAP Characteristics*

Patient characteristics	
Total no.	247
Male sex	159 (64%)
Age in yr	
18-20	1 (0%)
21-29	6 (2%)
30-39	16 (6%)
40-49	32 (13%)
50-59	75 (30%)
60-69	74 (30%)
70-79	33 (13%)
80-89	6 (2%)
≥90	0 (0%)
Prefer not to say	2 (1%)
No reply	2 (1%)
Height *(cm)*	178 (170-183)
Weight *(kg)*	83 (72-92)
Amputation characteristics	
Year of amputation	2011 (1993-2016)
Amputation level and side	
No amputation	1 (0%)
Transfemoral	
Left	95 (38%)
Right	87 (35%)
Transtibial	
Left	44 (18%)
Right	20 (8%)
Primary cause of amputation	
Trauma	156 (63%)
Oncology	40 (16%)
Infection	29 (12%)
Vascular	17 (7%)
CRPS	5 (2%)
BAP characteristics	
Year of BAP surgery	2019 (2017-2021)
BAP medical center	
RUMC	128 (52%)
OCR	119 (48%)
BAP treatment phase	
Waiting list for BAP surgery	26 (11%)
With BAP <12 mo	42 (17%)
With BAP 12-24 mo	41 (17%)
With BAP >24-36 mo	56 (23%)
With BAP >36 mo	82 (33%)

* BAP = bone-anchored prothesis, CRPS = complex regional pain syndrome, OCR = Osseointegration Center Rotterdam, and RUMC = Radboud University Medical Center. The values are given as the number, with the percentage in parentheses, or as the median, with the 25th and 75th percentiles in parentheses. The values for height and weight as well as percentages are rounded.

### BAP and Survey Evaluation

**Fig. 4:**
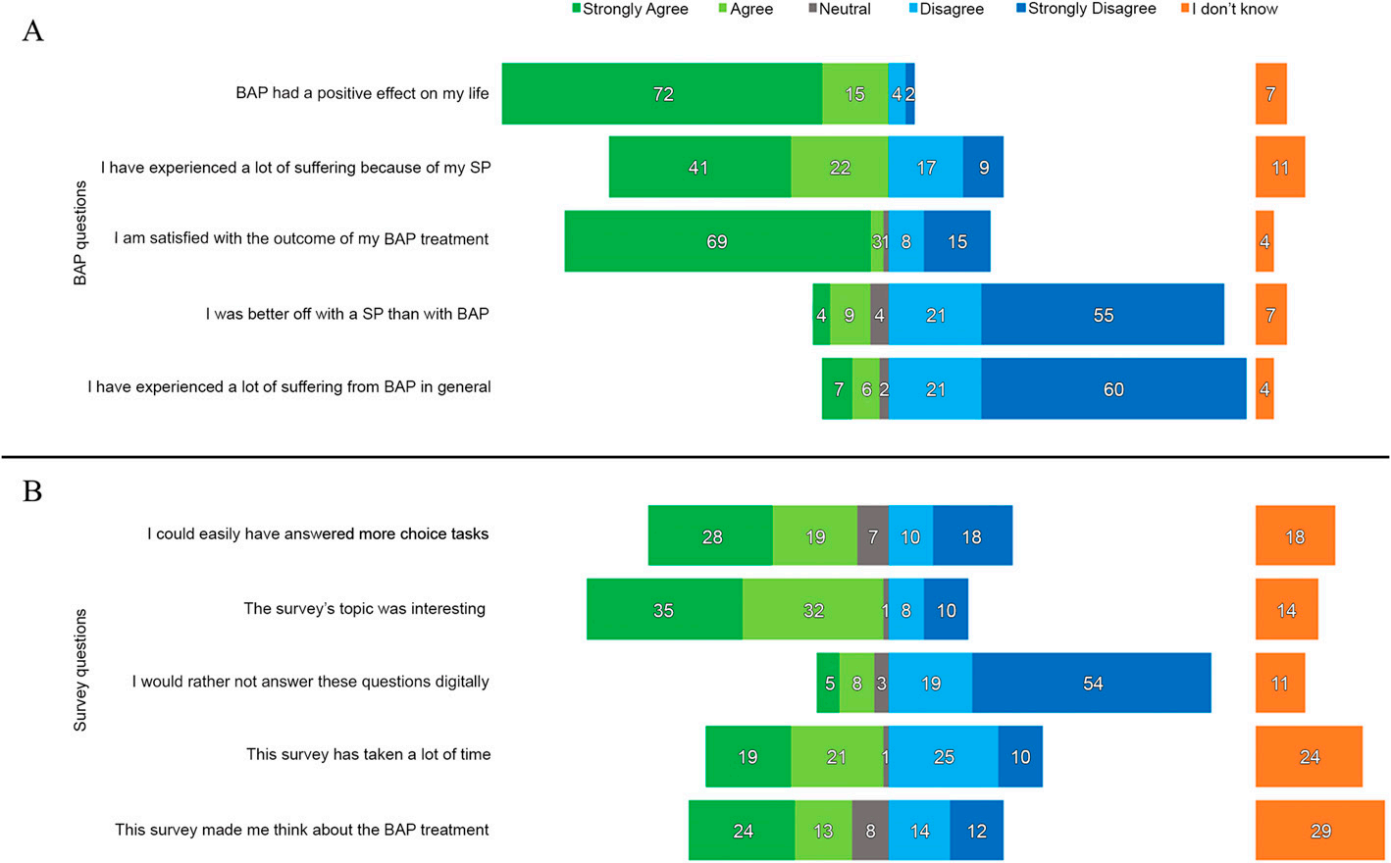
Percentages of agreement/disagreement with statements regarding bone-anchored prosthesis (BAP) treatment (**Fig. 4-A**) and the survey (**Fig. 4-B**). SP = socket prosthesis.

Seventy-two percent strongly agreed and 15% agreed with the statement, “BAP has had a positive effect on my life,” and 60% strongly disagreed and 21% disagreed with the statement, “I have experienced a lot of suffering from BAP in general” (Fig. 4-A). Regarding the survey statements (Fig. 4-B), 28% strongly agreed and 19% agreed that they “could easily have answered more choice tasks,” and 35% strongly agreed and 32% agreed that “This survey’s topic was interesting.”

### Preference Weights

**Fig. 5:**
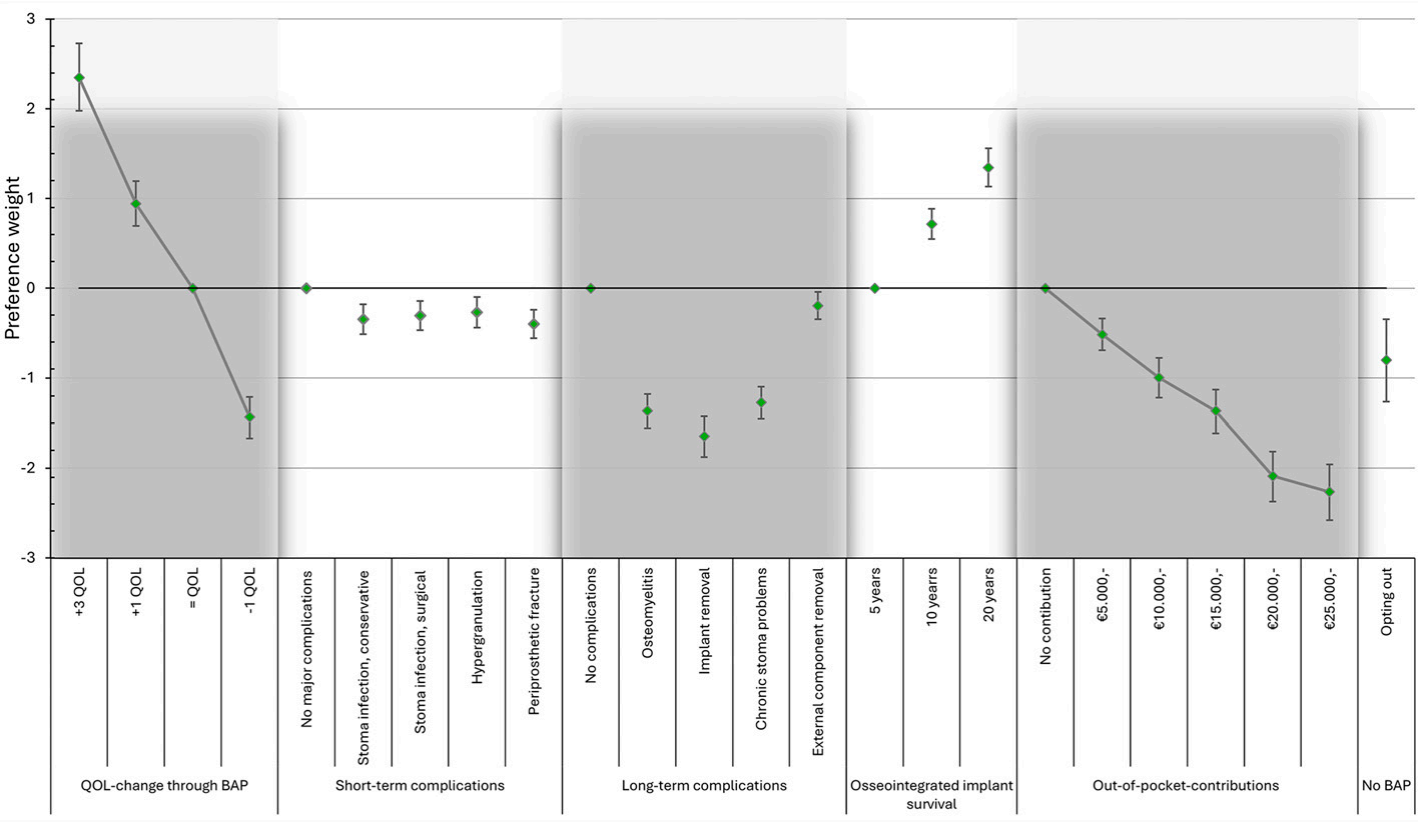
Mean preference weights for bone-anchored prosthesis (BAP) characteristics and levels. The whiskers indicate the 95% credible interval. QoL = quality of life.

Figure 5 provides the mean preference weights for all levels, which were significantly related to patients’ choices (as the 95% credible interval [CI] did not include 0), were in the expected direction, and adhered to the natural ordering of the levels for each characteristic, i.e., better outcomes had larger preference weights (also tabulated in Appendix S4). We found that patients would rather opt out of BAP treatment (−0.80; 95% CI, −1.26 to −0.34) when the BAP treatment would result in a 1-point decrease in QoL (−1.44; 95% CI, −1.67 to −1.21), as the negative preference weight was greater for the latter (−0.80 versus −1.44); otherwise, patients would not opt out of BAP treatment. Of all complications, implant removal had the strongest negative preference weight, −1.65 (95% CI, −1.88 to −1.43). Patients had a dislike of any out-of-pocket contributions. Given the patients’ preferences, the ideal BAP treatment would result in a 3-point QoL increase, and 20-year implant survival without any complications or any out-of-pocket contributions.

### Characteristic Importance

**Fig. 6:**
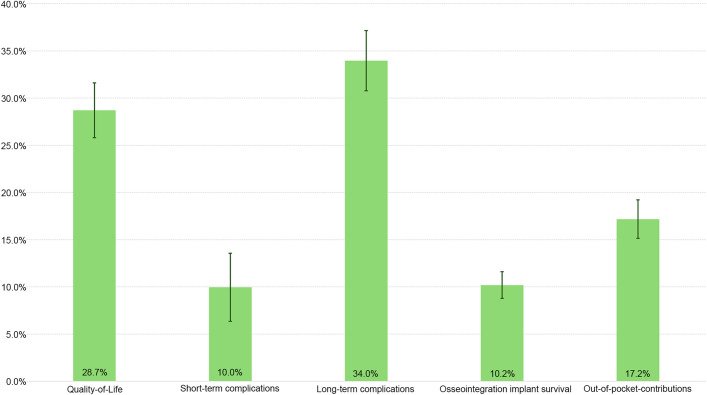
Relative characteristic importance by percentage (total = 100%). The whiskers indicate the 95% credible interval.

An analysis of overall characteristic importance indicated that long-term complications were most important (34.0%; 95% CI, 30.8% to 37.2%), followed by QoL (28.7%; 95% CI, 25.8% to 31.7%) and out-of-pocket-contributions (17.2%; 95% CI, 15.1% to 19.3%) when opting for BAP treatment (Fig. 6). Long-term complications were, on average, 3.4 times more important than short-term complications.

### Complications in QoL Points and Monetary Terms

For short-term complications, the level with the highest mean loss was periprosthetic fracture, at 0.28 (95% CI, 0.17 to 0.40) QoL points and €3,950 (95% CI, €2,180 to €6,130) (Table III). For long-term complications, the level with the highest mean loss was implant removal, at 1.15 (95% CI, 0.96 to 1.38) QoL points and €16,940 (95% CI, €14,780 to €19,040).

**TABLE III:** Complications as Expressed in Loss in QoL Points and Monetary Terms*

Characteristic	Level	Mean Loss (95% CI)	
		QoL *(points)*	Monetary† *(euros)*
Short-term complications	Stoma infection, conservative therapy	0.24 (0.12-0.37)	3.45 (1.67-5.65)
	Stoma infection, surgical therapy	0.21 (0.1-0.33)	3.05 (1.35-5.23)
	Hypergranulation	0.19 (0.07-0.31)	2.69 (0.93-4.92)
	Periprosthetic fracture	0.28 (0.17-0.4)	3.95 (2.18-6.13)
Long-term complications	Osteomyelitis	0.96 (0.79-1.16)	14.63 (11.32-16.90)
	Implant removal	1.15 (0.96-1.38)	16.94 (14.78-19.04)
	Chronic stoma problems	0.89 (0.73-1.08)	13.64 (10.08-16.25)
	External component replacement	0.14 (0.03-0.24)	1.94 (0.4-3.83)

* QoL = quality of life, and CI = credible interval.

† Values of €1,000.

## Discussion

This is the first study, to our knowledge, to investigate patients’ preferences for BAP treatment. Patients placed noteworthy importance on long-term complications, considering them the most crucial BAP characteristic. Patients also strongly cared about QoL and out-of-pocket contributions. This study also showed that patients had sound and logical considerations that followed the natural order of different levels.

To our knowledge, no previous DCEs have explored patients’ stated preferences for BAP treatment. However, in another DCE study, patients with a lower-limb amputation stated that minimizing costs was an important aspect of their treatment^35^. Therefore, as those patients and our patients both valued reduced out-of-pocket contributions, the estimates of our results are in line with those of the previous research^35^. Regarding complications, many studies concluded that risks and complications should be considered, despite positive clinical BAP outcomes^1-3,9^. In our study, patients were willing to accept risks given the improvement from a BAP when making a conjoint decision. As such, the behavioral patterns of patients undergoing BAP treatment are reflected in this study.

### Implications

By understanding what is important from the patient’s perspective, physicians may become more sensitive to the concerns of individual patients, which may have a positive effect on the process and outcomes of shared decision-making when comparing these results with the goals and preferences of patients who may be candidates for BAP treatment, i.e., management of patients’ expectations. This study underlines the importance of a patient-tailored approach for discussing BAP treatment and prognosis. By assessing patients’ experiences and preferences and providing quantitative data, it may have a positive effect on the decision-making process, including BAP information sessions and the informed-consent process, with respect to outcomes for individual patients and potential complications. Furthermore, the data can be used in health-technology assessments by incorporating patients’ stated complication-related loss in QoL points and cost. Lastly, patients can be informed numerically of the effect of a possible complication according to other patients with a BAP.

### Strengths and Limitations

This study used state-of-the-art DCE techniques to elicit preferences from patients across the whole spectrum of the BAP population (irrespective of amputation etiology and level and with different experiences with a BAP [in terms of both time and satisfaction]), which is important for generalizability. We also actively invited all patients by mail and phone to participate, even if they had an unpleasant experience, to reduce possible selection bias. Data quality is a strength, too. A post-hoc analysis, using a garbage class mixed logit, was conducted to evaluate the quality of the collected choice data^36^. Only 3% (n = 7) provided responses to the choice options that did not follow the theoretical foundation of utility maximization.

A limitation to this study is that all patients had experienced socket-related issues and therefore had, to some extent, incentives to choose a BAP in the DCE. However, some patients, from our experience, also opt out of BAP treatment after the information session on this treatment and accept their state without an osseointegrated implant. Thus, preferences given here are based on well-informed patients deciding to opt for a BAP given potential outcomes and risks. Another limitation is that we present the average preference for all patients, while accounting for preference heterogeneity. Additional analyses to identify different preferences are needed but were beyond the scope of this paper and will be conducted subsequently. Preference heterogeneity could be based on subgroups with different preferences in general or on background characteristics (e.g., age, sex, amputation etiology and level, experience with a BAP, implant type). The standard deviation describes the degree of preference heterogeneity per level (see Appendix S4). Note that this study included patients who experienced socket-related issues and who were eligible for, or have undergone, BAP treatment. Therefore, extrapolation to SP users without socket issues, or comparisons between those with and without socket-related issues, is not warranted. Given that 34% to 63% of SP users have reported having socket-related issues, the potential BAP cohort is much bigger^6,26,28^. Lastly, BAP treatment is currently offered in high-income countries to patients with socket-related issues. Whether our cohort had preferences similar to those of BAP cohorts elsewhere was not investigated here.

### Conclusions

To our knowledge, this study is the first to present patients’ preferences for use in discussing the treatment, outcomes, and related complications in shared decision-making regarding BAP treatment. The results suggest that osseointegrated implant teams and policy-makers should consider these areas when proposing treatment protocols. Furthermore, policy and clinical guidelines for BAP treatment could be enhanced by our results with respect to patients’ perspectives, management of patients’ expectations, and associated losses in QoL points and monetary loss secondary to complications.

## Appendix

Supporting material provided by the authors is posted with the online version of this article as a data supplement at jbjs.org (http://links.lww.com/JBJS/I177).
